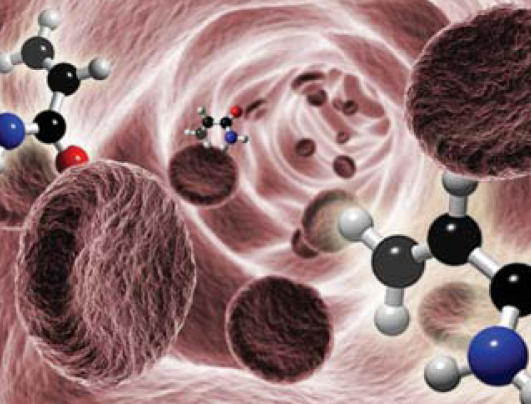# Diet & Nutrition: Acrylamide Study Suggests Breast Cancer Link

**Published:** 2008-04

**Authors:** Carol Potera

The International Agency for Research on Cancer classifies acrylamide as a probable human carcinogen. It has only been in recent months that an epidemiologic study first found a link between dietary acrylamide and human cancer risk. Now Danish researchers report that acrylamide adduct levels in blood are associated with an increased risk of breast cancer in postmenopausal women.

This is the first epidemiologic study to use blood biomarkers to assess acrylamide exposure. The findings, says first author Pelle Thonning Olesen, emphasize the importance of using biomarkers for exposure assessment. “Biomarkers are a more trustworthy indicator for exposure,” he says.

Before 2002, people were known to be exposed to acrylamide in certain industries and through smoking tobacco. That year, the Swedish National Food Administration discovered that acrylamide also forms in fried or baked starchy foods such as french fries, coffee, and baked goods. Diet is now thought to be the major source of exposure among nonsmokers, but the cancer risk posed by acrylamide in food is unknown.

All previous epidemiologic trials estimated acrylamide consumption from food frequency questionnaires. Olesen, a toxicologist at the Technical University of Denmark, Søborg, and colleagues instead measured levels of acrylamide and a key metabolite, glycidamide, bound to hemoglobin. The subjects included 374 postmenopausal women with breast cancer and 374 controls who participated in the Danish Cancer Society’s Diet, Cancer, and Health Study.

Adduct levels of acrylamide among smokers reflect both dietary and smoking intake of the compound. After statistical adjustments for smoking behavior, women with the highest acrylamide-hemoglobin levels showed a 2.7 times higher risk of estrogen receptor–positive breast cancer compared with women with the lowest acrylamide-hemoglobin levels. The risk rose with increasing acrylamide exposure.

Acrylamide-hemoglobin levels were not linked to estrogen receptor–negative breast cancer, and glycidamide-hemoglobin levels showed no connection with any breast cancer. The finding that only acrylamide-hemoglobin was associated with breast cancer suggests that the compound may induce cancer by nongenotoxic routes such as alkylation of proteins that could alter estrogen receptor function. The findings were reported in the 1 May 2008 issue of the *International Journal of Cancer*.

“This is an important study because it’s the first to measure acrylamide adducts,” says epidemiologist Lorelei Mucci, an assistant professor at the Harvard School of Public Health. Nonetheless, the study should be repeated in larger numbers of nonsmoking women, according to Mucci, because more than half the cases and controls were current or former smokers.

“The big public health question here is whether the amount of acrylamide in foods is enough to lead to cancer,” Mucci says. It is possible that other chemical compounds formed along with acrylamide may be the culprit in any cancer link. “Acrylamide-hemoglobin may be a biomarker for other carcinogenic chemicals formed during the heating of foods,” cautions Olesen.

## Figures and Tables

**Figure:**